# Adiponectin in psoriasis and its comorbidities: a review

**DOI:** 10.1186/s12944-021-01510-z

**Published:** 2021-08-09

**Authors:** Bai Ruiyang, Adriana Panayi, Wu Ruifang, Zhang Peng, Fu Siqi

**Affiliations:** 1 Department of Dermatology, The Second Xiangya Hospital, Central South University, Changsha, 410011 Hunan China; 2 Division of Plastic Surgery, Department of Surgery, Brigham and Women’s Hospital and Harvard Medical School, 75 Francis St., Boston, MA 02115 USA

**Keywords:** Adiponectin, Comorbidity, Immunity, Psoriasis, T lymphocytes

## Abstract

Psoriasis is a chronic, immune-mediated inflammatory skin disease characterized by abnormal T cell activation and excessive proliferation of keratinocytes. In addition to skin manifestations, psoriasis has been associated with multiple metabolic comorbidities, such as obesity, insulin resistance, and diabetes. An increasing amount of evidence has highlighted the core role of adipokines in adipose tissue and the immune system. This review focus on the role of adiponectin in the pathophysiology of psoriasis and its comorbidities, highlighting the future research avenues.

## Introduction

Psoriasis is a chronic skin inflammation involving hyperkeratosis and parakeratosis. It usually manifests as well-defined, raised, red plaques covered by silvery scales [1, 2] (Table 1). In the latest global epidemiological survey, the prevalence of psoriasis varied from 0.14% in East Asia to 1.99% in Australasia. The prevalence of psoriasis was also high in Europe and America [7]. The prevalence of psoriasis in Europe ranges from 0.6 to 6.5% and 3.15% in the US [8]. In China, the prevalence of psoriasis ranges from 0.44 to 0.54%, with 97% of patients having psoriasis vulgaris and 28% of patients reporting a family history of psoriasis [9] (Table 2). The relationship between psoriasis incidence and age appears to be relatively linear [12]. Psoriasis, mediated by T cells and dendritic cells, is closely related to the immune system and various clinical manifestations represent the activation of different components of the immune system. The adaptive immune system is mainly involved in chronic plaque psoriasis, while innate immune and autoinflammatory reactions play key roles in generalized pustular psoriasis [16]. Psoriasis vulgaris is considered one of the easiest human diseases to study [17]. Patients with psoriasis have a higher incidence of severe infections than those without psoriasis [18]. Furthermore, it has been shown that psoriasis itself may affect other organs [19]. For example, psoriasis is an important predictor of advanced liver fibrosis, which is not linked to age, sex, body mass index (BMI), hypertension or diabetes [20].

**Table 1:** The feature of different types of psoriasis [3–6]

Types of Psoriasis	Feature
Plaque psoriasis	Sharply circumscribed, round-oval, or nummular (coin-sized) plaques. The lesions may initially begin as erythematous macules (flat and 1 cm) or papules, extend peripherally, and coalesce to form plaques of one to several centimeters in diameter. A white blanching ring, known as Woronoff‘s ring, may be observed in the skin surrounding a psoriatic plaque.
Guttate psoriasis	Acute onset of a myriad of small, 2–10 mm diameter lesions of psoriasis. These are usually distributed in a centripetal fashion, it can also involve the head and limbs. Classically, it occurs shortly after an acute β-haemolytic streptococcal infection of the pharynx or tonsils and can be the presenting episode of psoriasis in children or adults occasionally.
Pustular psoriasis	Multiple tender sterile pustules with an underlying, blotchy, erythematous base. The patient may be pyrexial.
Erythrodermic psoriasis	Generalized erythema involving the majority of the body surface area. It may be a manifestation of unstable psoriasis precipitated by infection, tar, drugs, or withdrawal of corticosteroids.

**Table 2:** The incidence of psoriasis in all ages in different countries

Study	Country	Study period	Incidence rate per 100,000 person years (95% CI)
Bell et al. (1991) [10]	USA	1980–1983	59.9 (49.5 to 70.3)^a,b^
Donker et al. (1998) [7]	Netherlands	1995	120.0 (70.0 to 190.0)^a,b^
Jacob et al. (2016) [11]	Germany	2007–2010	521.1^a^
Egeberg et al. (2017) [12]	Denmark	2012	151.2 (148.0 to 154.5)^a^
Springate et al. (2017) [13]	UK	2013	129.0 (126.0 to 133.0)^a,b^
Kubanova et al. (2017) [14]	Russia	2016	65.0^a^
Schonmann et al. (2019) [15]	Israel	2017	276 (270 to 281)^a,b^

^a^Value reported from the study

^b^Age or sex adjusted

Adipose tissue participates in many physiological and metabolic processes in the body and acts as an energy reserve and an endocrine organ. Although fat cells are the main component of adipose tissues, they also include immune cells (such as T lymphocytes and macrophages) [21]. Previous studies have reported that adipose tissue has important immune functions and may be a major source of pro-inflammatory mediators, which promote the development of chronic inflammation, insulin resistance and atherosclerosis, all of which are associated with metabolic abnormalities [22]. The concept of ‘adipokine’ was first introduced when leptin was found in white fat [23]. By producing adipokines, especially adiponectin, adipose tissue is involved in many metabolic processes, including energy storage and temperature regulation. Adiponectin can also act on monocytes by regulating the polarization and proliferation of macrophages, thereby actively participating in immune responses and reducing T-cell reactivity and B-cell lymphocyte production [24]. Members of the C1q/TNF-related protein (CTRP) family, including adiponectin, play roles in metabolism and immunity [25]. A recent study suggested that adiponectin may also be a lipid carrier. Adiponectin and other CTRPs selectively bind to several anionic phospholipids and sphingolipids via the C1q domain in an oligomerization-dependent fashion, functioning as lipid opsonins [26]. AdipoR1 and AdipoR2 were identified as receptors for adiponectin. They are ubiquitously expressed, but AdipoR1 is more highly expressed in skeletal muscle, while AdipoR2 is more restricted to the liver [27]. Once bound to its receptors, AdipoR1 and R2, adiponectin initiates a series of tissue-dependent signal transduction events, including phosphorylation of adenosine monophosphate protein kinase (AMPK) and p38 mitogen-activated protein kinase (p38 MAPK), and it increases peroxisome proliferator-activated receptor alpha (PPARα) ligand activity, thereby reducing inflammation in various cell types [28]. For example, by inhibiting the nuclear factor κB (NF-κB) signalling pathway, adiponectin has an anti-inflammatory effect [29]. In view of the structural similarities between adiponectin and the CTRP proteins, these receptors may also respond to other CTRP family members [30]. Adiponectin may form heterotrimers with CTRP2 and CTRP9, but these trimers can only be generated when all three proteins are co-expressed in the same cell [31].

Therefore, whether adiponectin plays a key role in psoriasis and the underlying mechanism should be investigated. This review discusses the relationship between adiponectin and psoriasis, including the role of adiponectin in comorbidities, and the potential role of adiponectin in preventing or slowing the progression of psoriasis and its related comorbidities.

## Adiponectin and psoriasis

### Serum adiponectin and psoriasis

Adiponectin is a 30 kDa monomeric glycoprotein comprised of an N-terminal signal sequence, a nonhomologous or hypervariable region, a collagenous domain containing 22 collagen repeats, and a C-terminal C1q-like globular domain [28, 32]. Previous studies have suggested that adiponectin may be involved in the pathogenesis of psoriasis [33, 34]. Adiponectin plays an anti-inflammatory role in keratinocytes and inhibits the production and activity of pro-inflammatory cytokines, including the interleukin cytokines IL-2, IL-6, IL-8, IL-17 and IL-22; tumour necrosis factor-α (TNF-α); and interferon-γ (IFN-γ) and IFN-g, while increasing the secretion of anti-inflammatory cytokines such as IL-10 [35–37]. In T lymphocytes, it was previously found that non-recombinant adiponectin inhibited the production of IL-17, IL-22, TNF-α and IFN-g in Hut102 cells, possibly through AdipoR1, and the expression of adiponectin R1 in psoriatic epidermis was decreased [38]. Most studies have shown lower adiponectin levels in psoriasis patients than in healthy controls [39–41]. In experiments with psoriasis patients and control groups, the reduction of adiponectin and IL-10 was the predominant feature, followed by changes in IL-2, IL-12 and IL-17 [42]. Patients with psoriasis have lower plasma adiponectin levels, which may worsen the severity of their skin lesions [43]. Previous documents from 10 years ago revealed that plasma C-reactive protein (CRP) levels are negatively correlated with plasma adiponectin levels, and a significant negative correlation between CRP and adiponectin mRNA levels was also observed in human adipose tissue [44, 45]. However, in subsequent research, adiponectin serum levels were also positively correlated with the sedimentation rate (SR) and CRP levels, which was somewhat puzzling. Previously, the importance of this relationship in the course of psoriasis was unclear, but now it has been demonstrated that plasma adiponectin decreases and CRP increases as metabolic diseases progress [44, 46, 47]. Therefore, the reduction in adiponectin levels is closely related to the occurrence of psoriasis. As an inflammatory factor, CRP participates with various cytokines in the immune response and may play an important role in promoting the development of psoriasis.

**Fig. 1:**
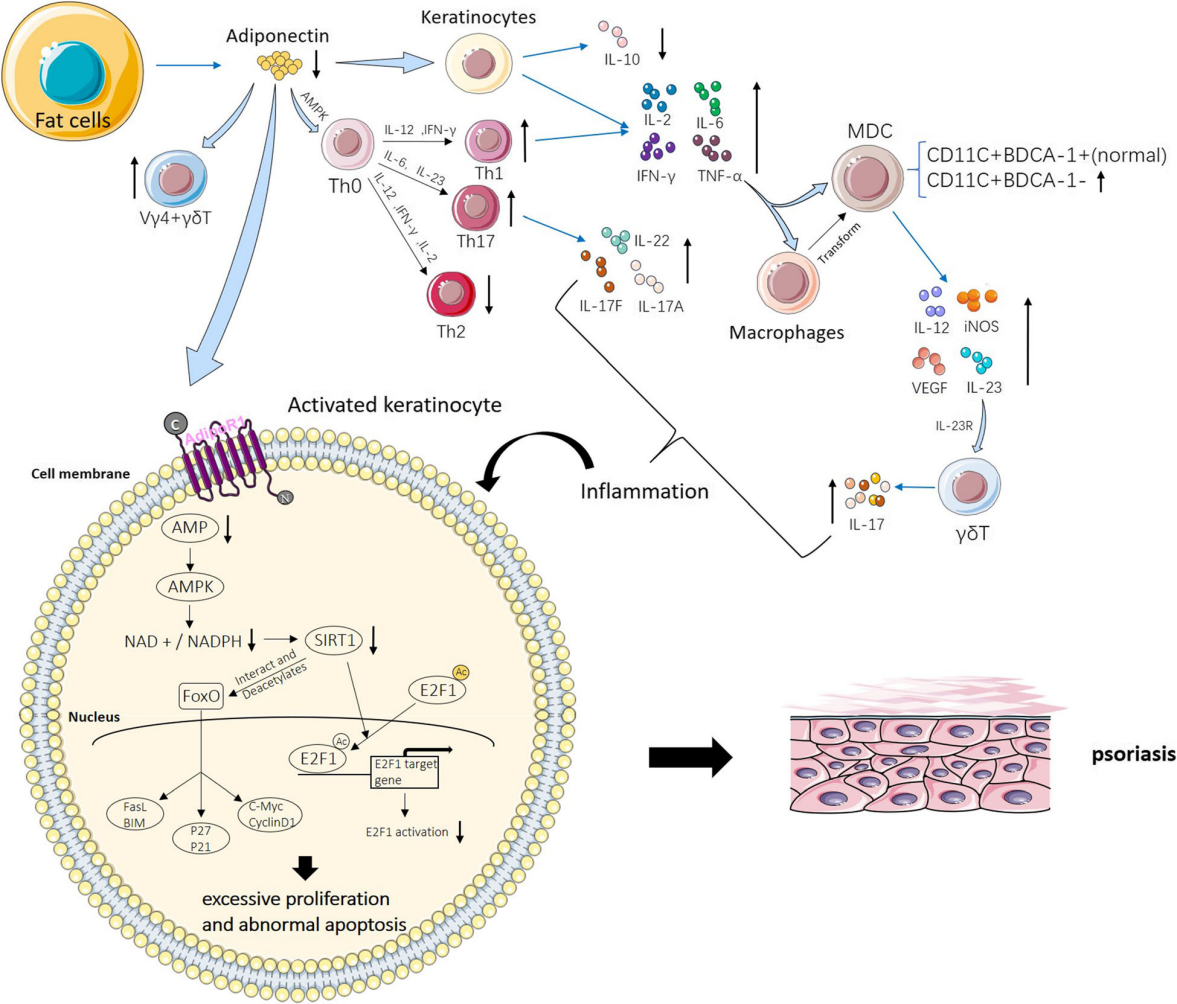
The role of adiponectin in the pathogenesis of psoriasis. Adiponectin is secreted by fat cells and can act on keratinocytes and naive T cells. A decreased adiponectin content leads to increased pro-inflammatory cytokines and decreased anti-inflammatory cytokines [60]. Among these, TNF-α can further affect macrophages and myeloid dendritic cells, resulting in increased secretion of cytokines [60]. In addition, adiponectin increases the number of Vγ4γδT cells. In keratinocytes, adiponectin can activate the E2F1 gene through the AMPK pathway, and the decrease in adiponectin levels leads to reduced E2F1 gene activation, thereby promoting the proliferation of keratinocytes [61–64]. In addition, SIRT1 can interact with the FoxO family. Downregulation of SIRT1 leads to abnormal transcriptional regulation of genes related to cell proliferation, survival, apoptosis, and metabolism, resulting in abnormal cell apoptosis [65–67]. These changes interact with the infiltration of the inflammatory response, which leads to psoriasis

Previous research on adiponectin focused on TNF-α and IL-6, which inhibit anti-inflammatory defences, particularly adiponectin levels [45]. Data from human and rodent in vivo studies have shown that adiponectin levels are negatively correlated with TNF-α and IL-6 [48–50]. Increased levels of pro-inflammatory cytokines, especially IL-6, in patients with psoriasis may be one of the causes of the decline in adiponectin levels in subcutaneous and visceral adipose tissue [51, 52]. Adiponectin can inhibit TNF-α [53], which can also be negatively regulated by TNF-α in psoriatic patients [54]. Anti-TNF therapy has also been found to decrease IL-6 levels in patients with psoriasis [55]. In addition, upregulation of anti-inflammatory cytokines helps restore the balance between Th (helper T cell) 1, Th17 and Th2 responses in patients with psoriasis [56]. In an AMPK-dependent manner, adiponectin inhibits Th0 cell differentiation into Th1 and Th17 cells [57]. Among its anti-inflammatory effects is inhibition of IL-17A production [58]. Adiponectin-deficient mice suffer from severe psoriasis-like skin inflammation and increased infiltration of IL-17 secreting skin Vγ4 γ δ-T cells, particularly upregulation of the Th17-related cytokines IL-17A, IL-17F and IL-22 [58]. Moreover, adiponectin also inhibits the synthesis and secretion of cytokine IL-17 by human CD4 (+)/CD8 (+) T cells [59] (Fig. 1). Consequently, adiponectin plays a key role in regulating psoriasis by directly inhibiting the secretion of IL-17 by T cells, which suggests new approaches to the study of psoriasis. Similar to adipocytes, sebaceous cells have also been found to differentially express and secrete adipokines. Adiponectin is expressed in human sebaceous glands (SGs), affecting the homeostasis of the dermis and promoting inflammation. Its relationship with systemic inflammation such as psoriasis is an interesting research pathway [68].

Subgroup and meta-regression analyses have found no significant correlation in terms of age, sex, psoriasis area and severity index (PASI) or study quality score with the expression level of adiponectin in psoriasis [39, 46]. This contrasts with previous findings that adiponectin levels are negatively correlated with PASI [54, 58, 69]. Despite the fact that most reports find the former [33, 50], there are indications that although the PASI score is negatively correlated with adiponectin levels, the correlation is very weak and therefore not clinically relevant [70]. Regarding the contradiction in the correlation between serum adiponectin and PASI, recent articles have shown that adiponectin is significantly reduced in the plasma of patients with mild (PASI< 10) or moderate (PASI< 20) forms of psoriasis, but it does not differ from controls in patients with severe psoriasis (PASI> 20) [46]. There are also articles suggesting that there is no correlation between adiponectin and PASI scores. This may be because most of the study subjects were patients with mild psoriasis, so a low PASI score indicates low disease activity with low TNF-a levels [71], meaning that the degree of correlation could be related to the patient sample selection. For the exact relationship between adiponectin and PASI, a single correlation cannot be used to discuss the overall relationship.

### Local adiponectin and psoriasis

There is increasing evidence that adipose tissue is locally involved in the pathophysiology of psoriasis and that dermal adipocytes participate in the steady-state temperature regulation of the skin [72–74]. In psoriasis, many different microorganisms play a pathogenic role, including *Staphylococcus aureus* [75]. Recent observations have found that after *S. aureus* infects the skin of mice, preadipocytes proliferate rapidly, and mice with impaired adipogenesis have a reduced protective effect against the infection, indicating that dermal adipocytes are also involved in skin protection against infection [76]. At the same time, skin without lesions in psoriasis demonstrates the same upregulation of cathelicidin expression after barrier disruption as the original psoriatic lesions, indicating that this response is local, not systemic [77].

Skin inflammation in patients with psoriasis is restricted to psoriatic plaques. This observation supports a primary local modification of the superficial layer of the subcutaneous white adipose tissue (sWAT) underneath the psoriatic plaques [72, 74]. It has been found that the local concentrations of adiponectin and TNF-α in sWAT were not significantly different between the control group and the psoriasis and/or metabolic syndrome group [73]. Another study found that the local adiponectin levels in the healthy skin and skin lesions of psoriasis patients were substantially lower than those in the control group [58]. The results of the former study may have been caused by the patients discontinuing hormone therapy before biopsy. This may have changed the local concentration of adipokines in the sWAT during the biopsy [73].

Ultrasound elastography can identify the involvement of subcutaneous adipose tissue in patients with plaque psoriasis and it can be used as an effective method to evaluate the early treatment response of patients with plaque psoriasis. The average strain ratio of the psoriasis lesion area was significantly higher than that of the non-lesion area [78]. Effective psoriasis treatment can greatly reduce the WAT hardness under the plaque, making it equivalent to the normal value of non-lesion skin, and this is related to an improvement in skin condition [79]. Therefore, the pathophysiology of psoriasis is not limited to the epidermis but should also include the spatially restricted tissue composite containing the skin and the adjacent WAT. Compared with changes in serum adiponectin levels, whether changes in local adiponectin levels have a greater impact on psoriasis is of interest.

## Adiponectin and psoriasis comorbidities

### Adiponectin and obesity

Psoriasis has been linked to obesity [80], especially abdominal obesity [81]. Obesity can also exacerbate existing psoriasis [82]. Abdominal obesity has been associated with a substantially low adiponectin level, demonstrated by a negative correlation between adiponectin level and waist circumference [71]. Obesity and psoriasis have a common pathogenic mechanism involving the fat cells critical for the synthesis of the pro-inflammatory cytokines IL-6 and TNF-α [42]. Because of the presence of these cytokines, macrophages infiltrate into the adipose tissue, and mature macrophages stimulate the secretion of cytokines, resulting in localized primary inflammation. Cytokines also trigger the production of inflammation-related proteins, leading to the low-level systemic inflammation observed in obesity [83, 84]. Obesity through pro-inflammatory pathways can induce the development of psoriasis and is a key factor in the progression of psoriasis in children [85]. Adiponectin levels decrease in obese patients [37, 86]. The diet-induced obesity model (DIO) has decreased adiponectin levels, while a high-fat diet (HFD) increases the frequency of T cells that are positive for IFN-γ and IL-17 [57, 87]. In early studies, meta- analyses showed no clear association between adiponectin levels and BMI [33], but later studies have shown that adiponectin levels are inversely proportional to BMI and low-density lipoprotein (LDL) and cholesterol levels [88–90]. In overweight or obese psoriasis patients, adiponectin levels have been observed to be significantly lower than in normal-weight psoriasis patients, but no hypoadiponectinemia was found [50, 70]. The high-molecular-weight adiponectin/total adiponectin ratio is decreased in patients with psoriasis [91]. Moreover, obesity is a predictor of poor treatment outcomes [92]. The use of methotrexate and anti-TNF treatment reduces the risk of comorbidities and is conducive to improvement of psoriasis [82], but anti-TNF-a antibody therapy is associated with an increase in adiposity. During anti-TNF-a treatment, the BMI of patients with psoriasis increase significantly [93]. The BMI increased by three points in patients treated with infliximab for 3 years [94]. Because of this adverse effect, some patients choose to terminate the treatment. Moreover, some studies claim to find no correlation between BMI and the severity of psoriasis [50, 70].

### Adiponectin and cardiovascular disease

There is biological plausibility for the link between psoriasis inflammation and cardiovascular disease because there is a common pathogenic mechanism. T cells have a well-defined contributory role to psoriasis. In atherosclerosis, naive T cells are known to play a pro-inflammatory role; once they migrate across the arterial lumen into the intima, they exhibit the characteristics of pro-inflammatory Th1 and Th17 cells [95, 96]. In addition, dyslipidaemia and atherosclerosis have been linked to reduced adiponectin levels [45, 97]. Adiponectin acts on endothelial cells, increasing local nitric oxide production, protecting endothelial function, and inhibiting plaque and thrombosis [98]. Nitric oxide (NO) derived from adipocytes can activate calcium-mediated potassium channel opening in vascular smooth muscle cells (VSMCs) and induce vasodilation [99]. Adiponectin also interacts with forkhead box O1 (FoxO1) to resist oxidative stress and inflammatory arterial damage by forming the FoxO1-C/enhancer-binding protein alpha transcriptional complex [100, 101] and inhibiting TNF-α-induced activation and the adhesion of monocytes to endothelial cells. Therefore, adiponectin may be significant in the prevention of atherosclerosis [102]. In terms of lifestyle, consumption of a HFD can cause an imbalance in the production of biomolecules around perivascular adipose tissue (PVAT), including increased TNF-α and IL-6 and decreased adiponectin levels, which in turn can lead to vascular dysfunction [103]. In high-risk populations with advanced atherosclerosis or other chronic inflammatory diseases, elevated adiponectin levels may be part of a compensatory mechanism that limits further endothelial damage [104, 105]. Patients with psoriasis receiving anti-TNF immune targeting and fumaric acid ester (FAE) have been shown to have a better prognosis [106, 107]. Not only does the PASI score improve, but adiponectin also increases, while high-sensitivity CRP decreases slightly [108]. Olive leaf extract can also significantly improve the expression of key adipogenic genes (such as PPAR, adiponectin and leptin receptors) in HFD-induced adipose tissue and can reverse the endothelial dysfunction observed in the aortic ring of obese mice [109]. This may be a promising new therapy for psoriasis and comorbidities. The role of adiponectin in conditions such as atherosclerosis and obesity has limited its use as a prognostic indicator for cardiovascular disease (CVD) in the general population [110–112] and as a biomarker for psoriasis patients with CVD [113].

### Adiponectin and diabetes

Psoriasis is associated with type 2 diabetes (T2D) [81]. Although diabetes is the least common component of metabolic syndrome, it is the most common cause of complications of advanced psoriasis [114]. Adipokines such as fetuin A and pro-inflammatory cytokines promote insulin resistance and disorders of energy and lipid metabolism [115]. In psoriasis, in addition to reducing adiponectin levels, fetuin A, may also contribute to excessive keratinocyte proliferation. Whether this is dependent on or independent of insulin desensitisation, it can further enhance metabolic syndrome and skin inflammation in psoriasis [116, 117]. Adiponectin levels in patients with psoriasis without diabetes are significantly higher than those of patients with progressive diabetes [70]. Substantially downregulated expression of adiponectin and its receptors in adipose tissues were also linked with diabetic dyslipidaemic conditions [47]. Experiments have shown that serum insulin levels and the insulin resistance index are positively correlated with the severity of psoriasis [118]. Adiponectin is also a significant regulator of the metabolic environment and energy balance, enhancing insulin sensitivity and reducing liver glycogen production [82, 119]. Therefore, lipid metabolism can be enhanced by increasing lipid clearance from the plasma, which can help improve blood glucose control [120]. In terms of treatment, anti-TNF immune-targeted therapy, such as adalimumab, and skin-directed therapy, such as ultraviolet B phototherapy, can reduce skin inflammation in patients with psoriasis, but adiponectin-induced glucose levels or insulin resistance do not change [55]. Systemic treatment with methotrexate results in a significant decrease in insulin levels and the insulin resistance index [118]. Pitavastatin has been shown to consistently increase plasma adiponectin, and it does not lead to new-onset or statin-induced diabetes [121, 122]. Perhaps statin therapy should be considered earlier in psoriatic patients with low adiponectin values [71].

### Adiponectin and other comorbidities

#### Non-alcoholic fatty liver disease

A correlation between non-alcoholic fatty liver disease (NAFLD) and psoriasis has been reported. Specifically, psoriasis patients have a higher probability of NAFLD [123], while the presence of NAFLD itself may exacerbate the severity of psoriatic skin lesions [124]. NAFLD is often seen in patients with early-onset psoriasis (74.2%), a diagnosis particularly common in patients younger than 40 years of age [114]. Adipokines play an important role in the pathogenesis of NAFLD, particularly adiponectin [115, 125]. Patients with psoriasis complicated by NAFLD have been shown to have lower adiponectin levels than patients with psoriasis alone [126, 127]. An in vivo study has shown that adiponectin deficiency not only increase the expression of NF-κB, but also promote the accumulation of lipid droplets and increase the production of reactive oxygen species (ROS), thereby aggravating liver damage and steatosis in an HFD-induced NAFLD mouse model [128]. In patients with psoriasis, it is reasonable to speculate that the reduction in adiponectin may play a role in promoting the development of NAFLD. In addition, increased expression of NF-κB has been demonstrated in psoriasis [129]. The increase in ROS cause Th1 and Th17 cells and keratinocytes to activate the MAPK, NF-κB and JAK-STAT pathways [130, 131], thereby forming a self-amplification cycle that ultimately leads to excessive keratinocyte proliferation, vascular proliferation and inflammatory infiltration, which is also characteristic of psoriasis [132].

#### Psoriatic arthritis

Psoriatic arthritis (PsA) and plasma adiponectin levels are negatively correlated [133]. A previous study found that the adiponectin level was significantly higher in PsA patients than in psoriasis patients without arthritis [69]. However, a study of patients with psoriasis with or without PsA found that adiponectin levels were not significantly different [70]. It is possible that most psoriasis studies do not appropriately control for parameters, such as the presence of PsA, which may contribute to the inconsistency of the results if the groups are not properly adjusted [73]. The effect of lifestyle has also been studied, and it was found that alcohol consumption obviously affects the negative correlation between adiponectin and PsA occurrence [133].

#### Mental disorders

Psoriasis increases the risk of mental disorders, including depression, anxiety and suicide [134]. In addition to the psychological stress caused by psoriasis, it has been reported that inflammatory cytokines are related to mood disorders, including elevated levels of cytokines, including TNF-a, IL-6 and IL-1 [135, 136]. Anti-depressants reduce the central nervous system’s inflammatory response and the levels of inflammatory cytokines, such as FAE [135, 137]. This not only improves the psoriasis and adiponectin levels but also efficiently reduces depression symptoms [107]. It can be speculated that the increase in pro-inflammatory cytokines caused by the decrease in adiponectin levels in patients with psoriasis may be one of the causes of depression.

## Adiponectin and anti-psoriasis treatments

In terms of treatment, secukinumab and etanercept have no relevant effect on adiponectin levels and they do not change the adiponectin level [70]. This may highlight that adiponectin levels are not closely related to PASI, a result consistent with other local and/or systemic treatment studies, except for analyses of secukinumab or other anti-IL-17 inhibitors [138, 139]. Patients using anti-inflammatory drugs or phototherapy may also have increased adiponectin levels [50, 140]. After mildly affected patients with no evidence of psoriatic arthritis received systemic methotrexate treatment, the PASI score was significantly reduced, but this treatment did not affect the serum adiponectin level [90]. During TNF-α treatment, an increase in adiponectin levels can also be observed [141]. Xue Li et al. found that hesperidin (Hes) can significantly improve psoriasis-like skin damage in mice and inhibit the proliferation of human immortalized keratinocytes (HaCaT) by inhibiting the activation of the IRS-1/ERK1/2 signalling pathway. After Hes administration, adiponectin levels in mice were significantly upregulated, and PASI scores were remarkably reduced. The secretion of pro-inflammatory factors in skin lesions, epidermal hyperproliferation and differentiation, and epidermal thickness were also reduced [142]. In addition, the level of IL-17A decreased significantly and the level of adiponectin increased significantly after treatment with Cal/BD aerosol foam. It is worth noting that there was a significant correlation between the improvement in PASI and the level of IL-17A [143]. Therefore, future research should examine the relationship between adiponectin and PASI and determine whether psoriasis can be treated by increasing adiponectin levels.

## Study strength and limitations

This review comprehensively summarizes the possible role of adiponectin as an immunomodulator and the correlation between adiponectin levels and various clinical parameters, emphasizing that adiponectin is likely to become a new target for the treatment of psoriasis.

## Conclusions

There is a complex relationship between psoriasis and fat. Studies on adipokines, particularly adiponectin, and their cytokine-related mechanisms are still insufficient, and information is lacking on whether these cytokines or their profiles can serve as biomarkers for psoriasis. Nevertheless, the importance of adiponectin in psoriasis is still of concern. With increasing research, the relationship between adiponectin and various systems of the body is becoming clearer, and changes in the expression of adiponectin can provide some guidance for patients with psoriasis regarding treatment. In the past, researchers have been more inclined to discuss systemic adiponectin, and the expression of local adiponectin in psoriasis may be more worthy of attention in the future. At the same time, it is also surprising that adiponectin may play a role as a lipid carrier, which provides a new direction for research. Currently, PASI is the gold standard for assessing the severity of psoriasis and its affected body regions, and the relationship between adiponectin and PASI should therefore be further clarified. In the evaluation of treatment, patient weight must be considered because there is a correlation between psoriasis and different comorbidities for which weight evaluation is valuable. The use of prior medications and the length of observation must also be considered, which may affect our judgement about the relationship between adiponectin and the course of psoriasis and even the evaluation of treatment efficacy. Furthermore, emphasis should also be placed on environmental and social factors involved in the disease, rather than focusing only on genetic and immune pathways. From a holistic perspective, a combination of biotherapy and measures to improve lifestyle can effectively alleviate the metabolic status of patients with psoriasis, including altering their adiponectin levels, which is indispensable for ameliorating the functional disorders of various systems in patients with psoriasis.

## Data Availability

Not applicable.

## Abbreviations

AMPK: Adenosine monophosphate protein kinase
p38 MAPK: p38 mitogen-activated protein kinase
PPARα: Peroxisome proliferator-activated receptor alpha
NF-κB: Nuclear factor κB
IL: Interleukin
TNF: Tumor necrosis factor
IFN: Interferon
SR: Sedimentation rate
CRP: C-reactive protein
SGs: Sebaceous glands
PASI: Psoriasis area and severity index
Hes: Hesperidin
HaCaT: Human immortalized keratinocytes
DIO: Diet-induced obesity
HFD: High-fat diet
BMI: Body mass index
LDL: Low density lipoprotein
VSMCs: Vascular smooth muscle cells
FoxO 1: Forkhead box O1
PVAT: Perivascular adipose tissue
FAE: Fumaric acid ester
CVD: Cardiovascular disease
T2D: Type 2 Diabetes
NAFLD: Non-alcoholic fatty liver disease
PsA: Psoriatic arthritis
NO: Nitric oxide
sWAT: subcutaneous White adipose tissue
CTRP: C1q/TNF related protein
ROS: Reactive oxygen species
